# Opportunities for deep learning techniques to advance the histological analysis of preclinical models of osteoarthritis beyond ordinal rank systems

**DOI:** 10.1093/jbmrpl/ziag039

**Published:** 2026-05-07

**Authors:** Jacob L Griffith, Yenisel Cruz-Almeida, Alejandro Almarza, Robert Caudle, Kyle D Allen

**Affiliations:** J. Crayton Pruitt Family Department of Biomedical Engineering, Herbert Wertheim College of Engineering, University of Florida, Gainesville, FL 32611, United States; Pain Research and Intervention Center of Excellence, College of Dentistry, University of Florida, Gainesville, FL 32603, United States; Department of Community Dentistry and Behavioral Science, College of Dentistry, University of Florida, Gainesville, FL 32603, United States; Department of Oral and Craniofacial Sciences, College of Dentistry, University of Pittsburgh, Pittsburgh, PA 15213, United States; Center for Craniofacial Regeneration, College of Dentistry, University of Pittsburgh, Pittsburgh, PA 15213, United States; Pain Research and Intervention Center of Excellence, College of Dentistry, University of Florida, Gainesville, FL 32603, United States; Department of Neuroscience, College of Dentistry, University of Florida, Gainesville, FL 32610, United States; J. Crayton Pruitt Family Department of Biomedical Engineering, Herbert Wertheim College of Engineering, University of Florida, Gainesville, FL 32611, United States; Pain Research and Intervention Center of Excellence, College of Dentistry, University of Florida, Gainesville, FL 32603, United States; Orthopedic and Sports Medicine Institute, College of Medicine, University of Florida, Gainesville, FL 32607, United States

**Keywords:** Analysis/Quantitation of Bone, Animal Models, Osteoarthritis, Bone Histomorphometry, Statistical Methods

## Abstract

Tools for assessing disease progression are needed to identify and confirm new mechanisms driving osteoarthritis (OA) progression and guide therapeutic development. This review focuses on the advantages and feasibility of leveraging deep learning techniques for quantitative analysis of rodent knee histology in preclinical OA models. Previous studies using radiographs and micro-CT have used deep-learning driven analysis, including convolutional neural networks (CNNs), to evaluate disease progression in OA patients and preclinical models. However, the use of these tools to analyze histology in rodent OA models is limited. The discrepancy between clinical and preclinical histological quantification partly relates to the size constraints of imaging, where the larger scale of clinical samples requires complex mapping and multiple samples to obtain a complete picture of the entire joint. Rodent OA samples provide a view of the entire joint on an individual slide and thereby enable the quantification of changes across the width of the joint. Here, we discuss approaches for using CNN-based pipelines to quantify and visualize joint remodeling in rodent OA models, complementing existing grading schemes and potentially providing insight into mechanisms driving joint pain and disability.

## Introduction

Histological analysis is a powerful tool for studying osteoarthritis (OA) in preclinical models, enabling deeper understanding of mechanisms driving OA pathophysiology. In preclinical models, the entire joint can be extracted. From here, traditional stains include toluidine blue, safranin-O with a fast green counter stain to visualize glycosaminoglycan content, and H&E to visualize structural and cellular changes in the joint. Additional techniques can be used to look at bone remodeling (bone histomorphometry) and the presence of specific immune cells (immunohistochemistry). Different stains can also be used on neighboring slides, which allows studies to overlap structural and cellular data within the same animals. Despite the attractiveness of understanding how these different variables interact, current approaches to analyze joint histology images are often inefficient and leave behind important spatial information.

For example, traditional histological assessments in preclinical OA models reduce complex joint remodeling information to ordinal rank scores. While these schemes are valuable for ensuring consistency of grading across laboratories and for comparing the overall magnitude of joint-remodeling, they present several significant limitations. Ordinal rank scores often fail to preserve critical spatial information (ie, specific location and distribution of cartilage degradation, subchondral bone changes, or synovial inflammation) across the joint surfaces. Furthermore, these categorical systems can struggle to discriminate subtle differences in joint structure that may be biologically significant in early disease stages or in response to therapeutic interventions. The reduction of continuous biological processes to discrete scoring bins (0-4 for the Krenn–Gu scheme or 0-6 for the OARSI scheme) inevitably results in the loss of sensitivity, particularly at the threshold boundaries between scoring categories.^1,2^ This is not criticism of ordinal rank scores. These systems are useful for standardizing scores across studies and laboratories; however, there are clear trade-offs between the relative strengths and weaknesses of ordinal rank-based, semiquantitative grading systems. Moreover, capturing spatial heterogeneity and fine details on structural changes may be crucial for understanding disease mechanisms and evaluating early intervention strategies in rodent models, particularly as we begin to integrate knowledge on other physiologic cues in the joint (innervation, vascularization, and distribution of immune cells throughout the joint, as examples).

To enable more detailed histological investigations, quantitative histological measures have previously been developed to complement traditional scoring systems. Many of these quantitative measures require manual segmentation (tracing each region of interest), which is both time consuming and tedious.^3,4^ This technical burden often limits sample throughput and introduces operator variability. Furthermore, the methodologies for measuring and reporting morphometric parameters frequently vary between studies and laboratories, hampering direct comparison of results across research groups. Addressing the shortcomings of current quantitative histological assessment systems could promote their wider adoption in the field. Moreover, improved efficiency, reproducibility, and standardization would significantly enhance the value of these techniques for evaluating disease progression and therapeutic interventions.

Deep-learning models offer a promising solution to streamline segmentations required for quantitative assessments of histology. Specifically, convolutional neural networks (CNNs), a powerful class of deep-learning architectures, have demonstrated remarkable success in complex segmentation and classification tasks across multiple domains.^5–9^ Within the context of OA, CNNs have even been used on a variety of imaging modalities to evaluate joint remodeling in patients and preclinical models. These applications include MRI and radiographic analyses of joint structures in clinical imaging data sets. However, despite this progress in clinical images of knee OA, their use in the quantitative assessment of joint histology remains limited. This represents a significant opportunity to develop automated approaches that could enhance throughput, reproducibility, and sensitivity of histological analyses in OA research.

This review focuses on the advantages and feasibility of integrating CNNs for quantitative analysis of rodent knee histology. Following a brief overview of OA-related histopathology, we describe current grading and analysis techniques. Additionally, we highlight opportunities to supplement existing ordinal rank systems with data from quantitative and spatial evaluations of OA. Since current segmentation methods to enable quantitative and spatial analysis are extremely time intensive, we next introduce and discuss how CNNs can be used to improve histological analysis in preclinical OA models. This begins with a general overview of CNNs and continues with a summary of current OA-related applications using CNNs in humans and preclinical models.

## Traditional pathology approach to preclinical models of OA

**Figure 1:**
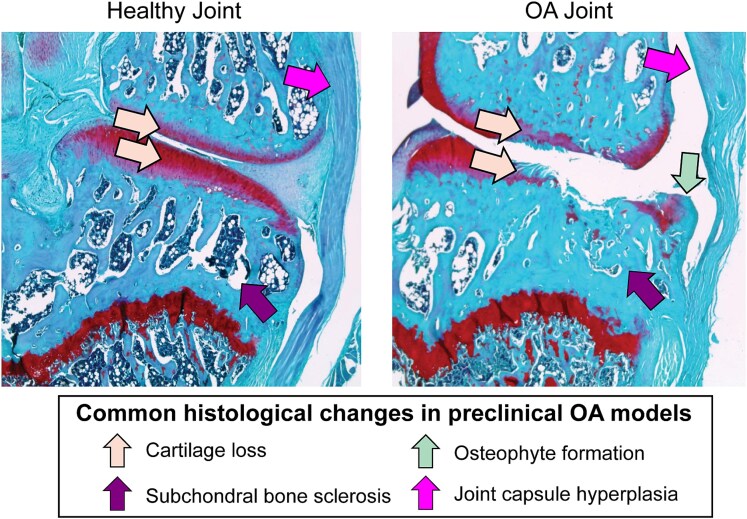
Osteoarthritis (OA) is associated with widespread joint remodeling including cartilage loss, subchondral bone sclerosis, osteophyte formation, and joint capsule hyperplasia.

Histological analyses provide crucial information about disease progression in preclinical OA models. Once considered a “wear and tear” disease centered on cartilage degeneration, OA pathogenesis is now widely accepted to change across the entire joint.^10–14^ Beyond cartilage loss, maladaptive remodeling occurs in the subchondral bone, including ossification of hypertrophic cartilage and formation of osteophytes. Moreover, chronic low grade inflammation occurs across the entire joint, including synovitis and joint capsule hyperplasia^2,10,15–18^ (Figure 1). The magnitude of joint remodeling is highly related to factors like age, sex, obesity, and the use of therapeutics, and understanding how these factors affect OA progression requires universal grading systems that enable results to be compared between studies and across laboratories.

To address this problem, conventional histological grading schemes have been developed to describe joint remodeling in preclinical OA models using ordinal ranks of disease severity. Ordinal scales are useful for summarizing complex information with an easier-to-interpret score that represents overall severity. This dimensionality reduction is attractive for describing complex joint-level changes related to OA. However, it is important to note that these scores are not traditional “numbers”; they are “ranks.” As with other rank-order data, a grade of 1 may be known to be lower than a grade of 2, and 2 lower than a grade of 3; but, the relative distance between each rank is not known. Mathematically, this means the distance between 1 and 2 may not be the same as the distance between 2 and 3. Thus, the order is known, but the distance between scores is not. Conceptually, this is akin to other ranking systems, like the ranking of some sports teams. The #1 ranked team may be better than the second or third ranked teams, but how much better is not known. In this way, ranks are ordered, but retain subjectivity. As an example, the Mankin grading system (or one of its modified versions), assigns a rank to articular cartilage structure, tidemark duplication, safranin-O staining, fibrocartilage, chondrocyte clones, hypertrophic cartilage damage, and subchondral bone thickness.^19–22^ A total score can be achieved by summing each parameter together. Moreover, to describe remodeling in different compartments of the joint, multiple Mankin scores can also be obtained.^22^ While this spatial information is valuable for understanding how preclinical models affect different regions within the joint, understanding the histological changes in the joint based on the score alone is difficult because there are multiple ways to obtain the same summed score. For example, 2 sections could receive the same overall Mankin score but have differences in subchondral bone or articular cartilage scores, and unfortunately, the sub-scores are often not reported.

In 2006, the Osteoarthritis Research Society International (OARSI) released standardized histological scoring guidelines.^23^ These guidelines introduced “principles for histological assessment” and provided a detailed grading scheme for the assessment of OA cartilage histopathology. These principles again aimed to guide development of new repeatable, easy-to-use analysis techniques. Additionally, the vision was to identify minimum reporting criteria, while giving flexibility to include complementary measures. The 2006 OARSI guidelines also provide updated and standardized definitions for OA cartilage histopathology features. Later, in 2010, OARSI released updated guidelines with species-specific recommendations that began to emphasize the utility of quantitative measures of cartilage damage, alongside the primary qualitative ranks.^15^ These updated guidelines again emphasized the importance of creating scoring systems that are intuitive and repeatable within and between observers. Furthermore, the 2010 OARSI histopathology initiative defined several variables that are important for histological analysis.^15^ The first definitions differentiated “staging” and “grading” of histology. “Staging” serves as an overall disease assessment, whereas “grading” is a more time-consuming process that gives detail on specific joint regions. The term “scoring” was coined to generally describe semiquantitative and quantitative evaluations, and “measuring” was defined as a specific quantitative evaluation of a specific OA-related feature. This distinction paves the way for new techniques focused on “grading and measuring” the effects of disease progression in preclinical OA models, and it is within the grading and measuring domain, where AI-based techniques can help to increase the throughput of samples and wide-spread use of these measures across the field.

## Integrating traditional OA scores and quantitative measures

Quantitative measures typically involve identifying regions of interest via segmentation and quantification (changes in shape or stain intensity via morphometry). The goal of introducing quantitative measures to evaluate histological changes in preclinical models of OA is not to replace ordinal grading schemes; instead, the goal is to provide additional tools that researchers can use to evaluate joint-remodeling. The OARSI 2010 guidelines for rat provided several examples of ways to implement quantitative measures that complement the ordinal rank scores, such as identifying and quantifying the width of cartilage loss at 0%, 50%, and 100% depth along with total tibial cartilage degeneration width.^15^ These measures showed high repeatability between both expert and novice graders. Our lab has also previously developed a graphical user interface for the evaluation of knee osteoarthritis (GEKO) to calculate morphometric measures of cartilage, subchondral bone, and synovium.^3,16^ Using GEKO, differences in post-traumatic OA models were compared to age-matched controls for measures like cartilage area, subchondral bone area, and joint capsule thickness.^3,16,24–27^ In addition, these segmentations enabled the correction of cartilage lesion morphometry maps that describe the shape and location of a cartilage lesion. The main limitation to these approaches is their tedious and time-intensive nature, even with graphic user interfaces. Therefore, harnessing technological advances to improve the efficiency of these systems is an opportunity to increase the usability and feasibility of quantitative histological analysis.

Examples of analyzing and visualizing spatial information can be seen in structural mechanics research. Although the mathematical methods may be more complex than what is needed for histological analysis, lessons can be learned from the emphasis placed on location-dependent information. For example, strain mapping is commonly used to overlay structural information with probabilistic heat maps of strain, allowing for visualization of the spatial variations of deformation within the structure.^28^ With spatial information on material properties, regional variations in stress can be approximated and visualized as well. Similarly, mapping probabilistic heat maps of joint remodeling in preclinical OA models with respect to joint structure provides a higher resolution of OA effects on joint structure. This could allow for deeper understanding of the spatial relationship between cartilage loss, subchondral bone sclerosis, and osteophyte formation. Furthermore, combining structural and spatial information with immunohistochemistry would provide deeper insights into OA pathophysiology and mechanobiological interactions between extracellular matrix structure and cellular changes.

A bottleneck to quantitatively and spatially evaluating histological images is current segmentation approaches. Although traditional image segmentation techniques, including thresholding, may be able to provide an automated approach for some subsets of images, histological variability between studies and labs often prevents successful widespread implementation of these approaches. As a result, manual segmentation (tracing each region of interest) is often necessary. While this segmentation approach can introduce inter- and intra- observer sources of variability, previous studies in the context of preclinical OA histology have shown such complications fall within an acceptable range. Although ongoing work is exploring potential advantages in repeatability and variability using CNN models, the potential for greater repeatability is apparent.^3,23^ Manual segmentation is both tedious and time intensive, taking over 5 min per image for complete segmentation of rodent knee samples.^3^ This problem is exacerbated when grading a large number of images across extensive and well-powered studies, exponentially increasing time burdens.

## A foundation for using CNNs in preclinical OA models

Computational pathology is an emerging field that leverages recent advances in artificial intelligence to digitally identify disease-related features in histological images.^29–31^ Within this space, CNN are emerging as a useful tool in computational pathology and biomedical imaging as a whole.^30,32,33^ This review explores their potential to advance computational pathology in preclinical OA models. To date, CNN-driven analysis in preclinical joint histology is minimal; however, CNN-driven pipelines have been used in clinical and preclinical studies to analyze CT and MRI data.

### Overview of CNNs

Convolutional neural networks are a powerful and robust class of deep-learning models capable of a variety of tasks, including segmentation and classification. These models learn to label regions of interest (segmentation) or assign a categorical value (classification) based on a set of features determined in the training phase. Unlike conventional classifiers, features are not defined a priori. In conventional classifiers, the usability of the system is largely dependent on the ability to define the correct features. Thus, a relationship between dependent and independent variables may appear non-existent without the “perfect” combination of features. By allowing the model to define features needed to complete each task, complex non-linear relationships can be more easily defined without introducing bias from user-selected features. Although an in-depth understanding of how CNNs work is not necessary to use these approaches, a basic conceptual understanding can be useful. For reference, a comprehensive description of CNNs can be found in.^34–37^ Based on their potential to enable objective, quantitative, and spatial evaluation of joint remodeling in preclinical models of OA, our discussion will focus on key concepts in CNN-driven segmentation.

**Figure 2:**
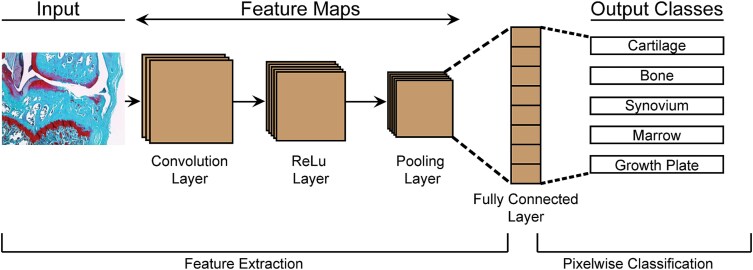
Representative diagram of convolutional neural network to segment histological images into regions of interest (output classes). In this model, convolution, ReLu, and pooling layers perform feature extraction and the fully connected layer connects to the output layer which classifies each pixel into one of the output classes.

For segmentation, CNN models use a supervised learning approach which requires a training set of images where regions of interest are known (ground truth). Using input and ground truth images, the model iteratively estimates segmentation (forward propagation) and adjusts these segmentations based on calculated performance metrics (backward propagation). Note, to best assess model performance, the trained model should be tested on an independent dataset. Convolutional neural network architectures consist of an assortment of input, convolutional, pooling, fully connected, and output layers^38^ (Figure 2). Convolutional layers pass a predefined kernel over the image to obtain features of the image including region geometry, color, and boundary detection. Pooling layers perform dimensionality reduction to allow for identification of low-resolution features by reducing “noise” in the image.^38^ Convolutional neural networks use fully connected layers to connect activations from convolutional and pooling layers to output layers which segment the image into the respective regions of interest based on pixel-wise classification.^38^ Thus, altering the number of layers and the order in which each layer type is arranged can improve CNN performance. Additionally, to improve performance, some CNN architectures add in dropout layers.^34–36^ These layers act as strategic checkpoints, where the model randomly omits some features used in forward propagation, forcing the CNN to find multiple paths to the same segmentation result.

To maximize segmentation and classification accuracy of the deep learning model, the sample size should be sufficiently large to capture the “population” variance. Thus, for widespread applications, the best training sets would include sections from multiple studies and labs (variability in staining and histological processing) and include both healthy and OA joints of varying severities (variability in region geometry). Training a CNN on a low variability sample will lead to overfitting of the model which can reduce segmentation accuracy when applied to images different from those in the test set, though these narrow studies will also likely be a starting point for implementing these technologies into the field at large. The primary method for avoiding overfitting is to increase variability of the training set by increasing sample size. However, if this is not possible, data augmentation can be an alternative. Data augmentation increases the number of images in the training set by using image transformations, such as rotations, contrast adjustment, and color normalization, thereby artificially creating an increased image variability.^34–36,39^ The types of data augmentations used should simulate variability that would occur due to variability in staining, histological processing, or imaging. Sources of such variability are common in histological analyses, including during tissue collection, sample preparation for sectioning, staining, and imaging. The method of tissue collection, including the storage medium, the type and length of tissue fixation, and for bony tissues, the type and length of decalcification processes, can have downstream effects during the staining process and represent a significant source of variation.^40–42^ The exact reagents used during staining, and sample time spent in each reagent, also frequently vary by protocol and institution. Variability originating from the selected imaging modality include in the chosen magnification, brightness, resolution, contrast, and background noise. Long-term, however, collaborative work throughout the preclinical OA research community would help to establish the necessary collection of histological images—capturing variability across multiple labs and studies—to train CNN models. This would allow for the training of relevant and well-fitted models with subsequent extensive external validation on a wide collection of images to verify reliability.

### Current applications of CNNs in OA

Clinically, a variety of novel and pre-existing CNN architectures have been applied to analyze radiographs in OA patients and healthy controls. Moreover, open-access data repositories have provided significant opportunities to achieve a “sufficiently large” sample size for developing CNN-driven techniques in clinical image datasets. For example, several studies have used data and radiographs from the Osteoarthritis Initiative (OAI),^43–55^ while a few other studies have pulled data and images from the Multicenter Osteoarthritis Study (MOST)^55–57^ and an outpatient clinic.^58^ For classification, CNNs have been designed to assign an ordinal rank score based on each radiograph, with multiple studies using CNNs to estimate Kellgren–Lawrence scores or related measures of OA severity.^44,46,47,51,54,55,57^ Here, CNN-based classifiers have even been shown to outperform traditional classifiers for hand-crafted features^44^ and were able to be classify knee OA with similar accuracy as a fellowship-trained arthroplasty surgeon.^58^ However, when evaluating the performance of CNN models, it is important to choose an appropriate metric. In classification tasks, prediction accuracy is typically used; however, Antony et al.^44^ suggests that mean square error is more appropriate. This justification is based on grading systems, such as the Kellgren–Lawrence scheme, being ordinal data instead of categorical. Therefore, the penalty for classifying a grade 1 image as grade 2 should be less than misclassifying a grade 0 image as a grade 4.

For segmentation, CNN-driven pipelines have been used in clinical image data sets to isolate the meniscus,^45^ cartilage,^48–50,52,53^ and bone.^50,52,53^ A majority of these investigations focused solely on segmentation and did not apply these pipelines to evaluate group differences between OA patients and healthy controls. However, Gatti and Maly^50^ and Tack et al.^45^ compared group differences between OA and healthy controls based on segmented cartilage and meniscus, respectively. Future work should build on the CNN-driven segmentations to establish utility for disease tracking and diagnosis.^59^ Clinical applications of CNNs in OA research provide examples for how preclinical researchers can implement these tools in studies focused on understanding OA pathophysiology.

While CNNs have been used in the categorization and segmentation of clinical images, preclinical applications of CNNs are rare. Current literature on CNN-driven analysis of histology in preclinical OA models is very limited with only 3 published manuscripts and 4 OARSI abstracts (to our knowledge). The first manuscript by Rytky et al. expands on their OARSI abstract from 2020.^60,61^ This study evaluated the application of CNN-driven segmentation to analyze calcified cartilage thickness in a rabbit model of OA in the medial and lateral compartments of the tibia and femur.^60^ Later, Mori et al. described a method for automating the detection of the medial and lateral compartments from histological sections of mouse knee joints.^62^ Although this approach does not quantify and evaluate joint-level changes in preclinical OA models, it shows feasibility of these approaches to identify regions of interest in the joint. Our own work has used CNNs to segment cartilage, bone, and marrow from the medial tibial plateau, and align these segmented regions to identify area of tissue remodeling in a rat OA model.^53^ The remaining 3 OARSI abstracts utilized the U-Net architecture to segment regions of interest in rat knees^63^ and elbows.^64^ Combined, this limited set of literature establishes feasibility and provides motivation for further implementation of CNNs for histological grading in preclinical models.

Beyond histology, work using CNN-driven approaches in OA preclinical models has also been applied to micro-CT images. Although these studies do not use histological images, the underlying premise is that CNNs streamline quantitative assessment of joint-level changes in OA. Combined with their work using histological sections, Rytky et al. also introduced CNN-driven approaches to segment and evaluate micro-CT images of calcified cartilage thickness in a rabbit model of OA.^60^ In mice, Mahdi et al. introduced a CNN-driven pipeline to automate micro-CT segmentation and microstructural analysis of tibial subchondral bone.^65^ This approach showed similar results to manual methods for bone volume, total volume, bone volume fraction, trabecular thickness, and bone surface density. However, trabecular number and trabecular separation values calculated with the CNN-driven approach were statistically different than those calculated using conventional methods. Although CNN-driven analysis of histology and micro-CT in preclinical models of OA has not been widely performed, the few existing studies provide a strong guide for future implementation of this technology.

## Conclusion

Integrating CNN-driven histological analysis in preclinical OA models offers a valuable complement to existing histological grading and scoring techniques. By increasing the amount of information captured, these new techniques can establish foundations for deeper understanding of joint-level processes. In traditional OA grading schemes, complex joint-level changes are often reduced to ordinal rank values. Although useful for comparison and repeatability, this prevents deeper interpretation of nuanced differences in various preclinical models of OA and for the assessment of emerging therapeutics. For example, the location of cartilage loss and subchondral bone sclerosis may vary between the anterior cruciate ligament transection and medical meniscus transection models and the location of the damage may relate to joint pain and other symptoms. Methods to intuitively visualize these nuanced differences do not currently exist, and by developing quantitative and location dependent analysis techniques via AI-based analyses, a deeper understanding of joint changes may be obtained, which could both decrease subjectivity and increase scientific rigor.

Overall, quantification and visualization of histological changes in preclinical models of OA can be performed with manual segmentation techniques, but the time intensive nature is a major bottleneck. Therefore, creating high-throughput pipelines to increase the efficiency of quantitative histological analysis and reduce the burden of segmentation would make implementation of these approaches across the field more attractive. Although the task of creating CNN-driven pipelines to analyze histological images in preclinical models of OA can sound daunting, current literature supports the feasibility of translating these approaches. Specifically, methods used to analyze OA progression in humans and preclinical models provide a decent roadmap to follow. For these efforts to be successful, collaboration is necessary. As seen with human studies using CNN-driven pipelines, libraries that combine images from multiple studies and institutions are useful for generating a sufficiently large sample size that can capture the variability of images. In the same way, segmentations from multiple experts would help to improve model training for complex disease features. Moreover, building a diverse library of images and grades by multiple experts is important to representing the entire field, essentially creating a preclinical resource that is analogous to the clinical data repositories seen with the OAI. Further, creating pipelines to segment and quantify joint remodeling alone should not be the only goal; to leverage these advances, the field may need to rethink how to visualize nuanced, spatial changes in the joint.

In conclusion, implementing CNN-driven pipelines to quantify and visualize joint remodeling in preclinical OA models can provide insight into mechanisms driving pain and disability. These approaches could increase the utility of preclinical models, while encouraging and rewarding collaborative research. Solving the mysteries of OA pathophysiology is not feasible by one study, or even one lab. Therefore, repeatable tools for assessing disease progression are needed to identify and confirm new mechanisms driving OA progression and lead to the comprehensive evaluation of disease modifying therapeutics in the future.

## Data Availability

The data that support the findings of this study are available from the corresponding author, KDA, upon reasonable request.
